# Pembrolizumab for high TMB castration-resistant prostate cancer: A precision medicine case report

**DOI:** 10.1007/s13691-025-00833-3

**Published:** 2026-01-12

**Authors:** Vincenzo Terrano, Erica Perri, Chiara Cioffo, Giusi Mastroianni, Maria Antonina Palumbo, Fortunato Ciardiello, Teresa Troiani, Stefania Napolitano

**Affiliations:** Department of Precision Medicine, Università Degli Studi Della Campania “Luigi Vanvitelli”, 80138 Naples, Italy

**Keywords:** Tumor mutational burden (TMB), Metastatic castration-resistant prostate cancer (mCRPC), Pembrolizumab, Immunotherapy

## Abstract

This case report describes the clinical management of a 68-year-old man with concurrent invasive melanoma and metastatic castration-resistant prostate cancer (mCRPC). Initially diagnosed with BRAF V600E-mutated melanoma, he received Dabrafenib and Trametinib. Later, prostate cancer was diagnosed with bilateral pulmonary metastases. Despite initial treatment with Triptorelin, Docetaxel, and Abiraterone, disease progression occurred. A liquid biopsy revealed an extremely high Tumor Mutational Burden (TMB) and mutations including *POLE, BRCA2, ATM, and TP53.* Due to the high TMB, off-label Pembrolizumab was initiated. Radiological evaluations at 3 and 6 months showed a marked response, with disappearance of target lung metastases and durable remission maintained through February 2025. Only grade 1 asthenia was reported, without significant treatment interruptions. This case illustrates the value of precision medicine and the role of liquid biopsy in guiding immunotherapy decisions for complex oncological cases. It supports the relevance of molecular profiling in selecting effective treatments beyond standard indications.

## Introduction

Prostate cancer is one of the most common neoplasms in men, often characterized by heterogeneous biology and, in advanced cases, complex therapeutic management. This complexity is magnified when there are concurrent oncological diseases, which pose significant challenges in terms of treatment sequencing, toxicity, and overall prognosis. In such scenarios, the choice of the most appropriate therapies—together with the integration of innovative diagnostic and therapeutic approaches—becomes crucial to optimize patient outcomes. We present the clinical case of a patient with a diagnosis of both invasive melanoma and metastatic prostate cancer, treated off-label with pembrolizumab, highlighting the importance of a multidisciplinary strategy, the use of liquid biopsy and personalized molecular therapy, and the role of comprehensive genomic profiling in the management of such complex cases. This case underscores how modern oncology increasingly relies not only on the standard algorithms of prostate cancer management (which increasingly include novel systemic therapies for advanced disease) but also on tailoring treatment to the unique molecular and immunological profile of each patient, particularly when dealing with multiple malignancies and overlapping therapeutic landscapes.

## Case report

### Initial diagnosis of melanoma

A 68-year-old man underwent an excisional biopsy of a melanocytic lesion on his right thigh on December 9, 2020. The lesion was diagnosed as an invasive melanoma. Breslow thickness: 2 mm. Ulceration: present. Mitosis: 3/mm 2. Microscopic satellitosis: absent. Regression: not evident. Tumor-infiltrating lymphocytes (TILs): absent. Perineural infiltration: absent. Neoplastic vascular embolism: absent. Margins of exeresis: absent. On February 4, 2021, he underwent a radical surgical procedure and a sentinel lymph node biopsy in the right inguinal region. The biopsy revealed the presence of melanoma metastases, classifying his stage as pT3bN1a, IIIC, with a *BRAF V600E* mutation [1]. After the diagnosis, the patient underwent a radiological staging through a total body computerized tomography (CT) scan and 18- Fluorodeoxyglucose (FDG) computerized tomography (CT)/positron emission tomography PET scan which revealed the presence of a single pulmonary lesion with increased metabolic grading.

### Targeted therapy and onset of urinary symptoms

Starting from April 2021, after discussion at the multidisciplinary group, considering the stage and molecular characteristics of disease, as well as the patients wish, he began first-line targeted therapy with Dabrafenib and Trametinib, a therapeutic combination chosen based on the presence of the *BRAF V600E* gene driver mutation [2]. In July 2021, the patient presented symptoms of dysuria and stranguria, leading to a urological visit and a multiparametric magnetic resonance imaging (MRI) of the prostate. Even though the prostatic specific antigen (PSA) value was negative, the examination revealed the presence of a large, heterogeneous solid tissue, classified as Prostate Imaging Reporting and Data System (PI-RADS) 5, indicative of a highly suspicious malignant lesion. Concurrently, a total body CT scan showed a doubling of the size of the pulmonary lesion and the appearance of new bilateral solid lesions. The patient’s history of *BRAF V600*–mutated melanoma and prior treatment with combined BRAF/MEK inhibition may also be relevant to the observed immunotherapeutic response. Beyond its direct antiproliferative effect on tumour cells, it is plausible that the prior dabrafenib/trametinib regimen indirectly contributed to the exceptional subsequent response to pembrolizumab by favorably modulating the tumour immune microenvironment [3]. Indeed, preclinical and clinical data in *BRAF*-mutant melanoma demonstrate that BRAF and MEK inhibition led to increased tumour-infiltrating lymphocytes (TILs), enhanced antigen presentation and reduced expression of immunosuppressive cytokines (such as IL-6 and IL-8) [3, 4]. These changes effectively convert an immunologically ‘cold’ tumour milieu into a more ‘hot’ and immune-responsive context. While we lack direct immunologic readouts (e.g., TIL quantification, PD-L1 expression) in our patient, the sequence of events – namely, BRAF/MEK targeted therapy, followed by a durable complete remission on anti-PD-1 therapy in a *POLE*-mutated ultramutated prostate cancer – supports the hypothesis of immune priming by the prior targeted regimen. This suggests that in exceptional responses to immune checkpoint inhibitors, oncologists should consider not only tumour genotype (e.g., *POLE* mutation, TMB high) but also the history of prior targeted treatments and their possible immunomodulatory impact. Future studies are needed to clarify whether prior MAPK pathway inhibition can synergize with immunotherapy and to better define the interplay between targeted therapy and immunotherapy.

### Diagnosis of metastatic prostate cancer

The case was discussed in the multidisciplinary oncology group, where, after reviewing the images, in view of the increased size of the right lung lesion, the patient was candidate for CT-guided fine-needle aspiration cytology (FNAC), in order to make the differential diagnosis. The pulmonary biopsy confirmed the presence of metastases from carcinoma, with a primary prostate origin.

### Molecular profiling through liquid and tissue biopsy

On August 24, 2021, the patient underwent a liquid biopsy using the Foundation One CDx test, an advanced genetic test approved by the Food and Drug Administration (FDA) to analyze circulating tumor DNA (ctDNA) in the blood. This test is particularly useful for identifying mutations, genetic rearrangements, and copy number alterations of specific genes related to various types of cancer. In this patients case, the test revealed an extremely high TMB of 421 mutations per megabase (Muts/Mb), likely due to the presence of the pathogenic P286R mutation in the *POLE* gene, specifically in the proofreading exonuclease domain.

In addition to this mutation, the patient had other significant genetic alterations involving key genes for melanoma and prostate tumors, including *BRCA2, ATM, CHEK2, PTEN, SPOP, PIK3CA, PIK3R1, TP53, RB1, ERBB2, NF1, and POLE,* while no *B-RAF* gene mutation was found to be ascribed to melanoma in history. These mutations are not specific to a single type of cancer, but their identification in the patients tumor profile can be crucial for guiding therapeutic decisions, especially in the context of targeted therapies and immunotherapy. The results of the liquid biopsy were supported by a tissue biopsy of the melanoma, which showed mutations in the *BRAF V600E, MTAP, CDKN2A/B, P2RY8, and TERT genes,* with a TMB of 5Muts/Mb. Moreover, MSI (microsatellite instability) status was assessed on the available prostate tumour specimen using next-generation sequencing (NGS) and microsatellite-loci analysis combined with mismatch-repair (MMR) gene evaluation. The tumour did not meet criteria for MSI-High (MSI-H) (for instance, MSIsensor score < 10, no detected deleterious MMR gene variants and no loss of MMR protein expression on immunohistochemistry). Thus, the tumour was classified as microsatellite stable (MSS) despite the ultramutated phenotype. This finding aligns with emerging evidence that pathogenic *POLE*-exonuclease-domain mutations can drive a high TMB through a mechanism distinct from classical MMR deficiency and MSI-H [5]. Indeed, in prostate cancer and other tumour types of *POLE*-mutated cases often show MSS status yet still demonstrate ultra-high mutational burden, suggesting that reliance solely on MSI-H status may underestimate immunogenic potential in such cases [5, 6].

### Histological and radiological staging

The prostate biopsy, on the other hand, confirmed the diagnosis of acinar adenocarcinoma with comedonecrosis, classified as grade 10 (5 + 5) according to the Gleason combined score and belonging to group 5 according to the ISUP/Epstein classification. A subsequent total body CT scan confirmed the presence of bilateral pulmonary metastases, while a bone scan did not reveal secondary lesions, thus excluding the presence of bone metastases.

### First- and second-line treatments

In October 2021, the case was newly discussed in the multidisciplinary oncology group, where, given the clinical stage, it was considered a priority to start first-line medical oncological treatment for prostate cancer, while the patient was referred for periodic clinical instrumental follow-up for melanocytic pathology. From November 2021 to March 2022, the patient underwent an initial oncological treatment with Triptorelin and Docetaxel, achieving a partial response to the therapies as evidenced by the RECIST criteria (Response Evaluation Criteria in Solid Tumors) [7]. However, in June 2022, due to disease progression in a pulmonary lesion, a second-line treatment was initiated, including Abiraterone, Deltacortene, and Triptorelin, integrated with stereotactic radiotherapy and radiotherapy on the prostatic bed [8].

### Disease progression and initiation of immunotherapy

In June 2023, the patient registered further progression of the pulmonary lesions, reducing the available therapeutic options, and requiring the identification of an effective and safe treatment. In response to this, considering the restricted therapeutic alternatives and the high TMB shown in the Foundation liquid biopsy result, off-label administration of Pembrolizumab was started [9, 10]. The choice of this treatment was supported by the results of the Keynote-158 study, which demonstrated the efficacy of Pembrolizumab in patients with advanced and metastatic tumors, especially those with MSI-H status and high-TMB who had already received previous treatments, excluding colorectal tumors. Among these patients, those with prostate cancer were also included, providing a solid basis for the use of this drug in similar clinical cases [11–13].

### Radiological response and current status

**Fig. 1:**
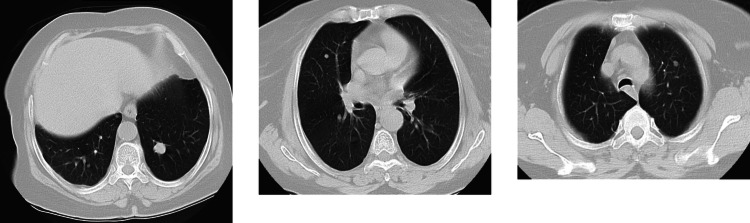
Sequential thoracic CT scans showing pulmonary metastases and treatment response. (**A**) Baseline CT (June 2023): multiple bilateral pulmonary metastases. (**B**) After 3 months of pembrolizumab (October 2023): partial reduction in lesion size. (**C**) After 6 months of treatment (March 2024): complete radiologic remission of lung lesions

After three months of treatment, a first radiological evaluation showed a partial response, with a reduction in the size of the pulmonary lesions. This positive response was confirmed by a second evaluation in March 2024, indicating no evidence of target metastatic lesions at the lungs. Successive instrumental evaluations using total body CT scans performed every 3 months, the last one in February 2025, showed the persistence of the beneficial effects of the treatment. During the period of treatment with Pembrolizumab, the patient reported only grade 1 asthenia, a minimal and easily manageable side effect. Despite this, the patient continued with the treatment without significant interruptions, maintaining a good quality of life and effective control of the disease Fig. 1.

### Follow-up

In September 2025, a FoundationOne CDx tissue biopsy was performed on an archival tumor specimen obtained at the baseline. The genomic profiling revealed a set of mutations that partially overlapped with those identified by the initial liquid biopsy — including *POLE P286R* and alterations in *ATM, CHEK2, PTEN, SPOP, PIK3CA, PIK3R1, RB1, ERBB2, NF1* — confirming a core molecular signature. Nonetheless, certain discordances were observed: notably, the *TP53* alteration and the *BRCA2* variant found in the liquid biopsy were not detected in the tissue‑based profile, possibly due to assay sensitivity differences or intratumoral heterogeneity. Further updates on the case are expected in the coming months.

## Discussion

This case demonstrates the efficacy of pembrolizumab in a patient with metastatic prostate cancer, emphasizing the importance of considering innovative therapeutic options even in off-label regimens. A multidisciplinary management and a personalized approach allowed for excellent treatment tolerance and effective disease control. Moreover, this case underscores the critical role of liquid biopsy and molecular therapy in the management of patients with multiple tumors: the liquid biopsy revealed an extremely high tumour mutational burden (TMB) and significant key mutations, which guided our therapeutic decisions toward a more personalized and effective strategy. The adoption of therapies tailored to the patient’s specific genetic profile significantly improved the clinical outcome, highlighting the promise of precision medicine in complex oncological settings. In the broader clinical context, checkpoint inhibition in MSI-high (MSI-H) or TMB-high prostate cancer — although uncommon — has shown meaningful activity: durable responses to pembrolizumab were observed in selected mCRPC patients with MSI-H—and even in some with TMB-high disease. Our case adds further evidence with a durable complete pulmonary response in a high-TMB mCRPC (driven by *POLE* mutation) and reinforces the value of comprehensive genomic profiling for optimal treatment selection (Table 1).

**Table 1:** Clinical studies of pembrolizumab in metastatic prostate cancer with MSI-H/dMMR or high TMB ^1^

Study	Setting/N	Biomarker	Treatment	Outcome
[14]	mCRPC (N = 1)	MSI-H/dMMR (somatic EPCAM, MSH2, MSH6 co-deletion)	Pembrolizumab	Exceptional response
[15]	mCRPC (N = 1)	MMR-deficient	Pembrolizumab	Durable response
[16]	mCRPC (MSI-H subset, N = 12)	MSI-H/dMMR	Pembrolizumab	PSA50 = 75%; ORR ~ 57% (radiographic)
[17]	mCRPC (N = 1)	MSI-H	Pembrolizumab	Partial response; PSA decline
[18]	t-NEPC (treatment-related neuroendocrine prostate carcinoma) in CRPC (N = 1)	High TMB (tumor mutational burden)	Pembrolizumab	~ 75% reduction of retroperitoneal mass
[19]	mCRPC (N = 1)	MSI-High & TMB-High	Pembrolizumab	Marked PSA reduction and shrinkage of metastases

**Notes:** a. ORR = overall response rate; PSA50 = prostate-specific antigen decline ≥ 50%.

b. Some studies include single-case reports; sample sizes are small.

c. Treatment lines prior to pembrolizumab vary across studies.

## Materials and methods

Clinical data collection Clinical and pathological data were retrospectively retrieved from medical records, imaging studies, histological reports, and multidisciplinary tumor board discussions. The case was managed at a tertiary referral academic center, and all diagnostic and therapeutic procedures were performed according to standard clinical practice.

### Histopathological diagnosis and staging

Melanoma diagnosis and staging were based on the AJCC 8th edition criteria. Prostate cancer was diagnosed as acinar adenocarcinoma and classified according to the ISUP/Epstein grading system. Gleason scoring was performed on biopsy specimens. No bone metastases were detected on bone scintigraphy.

### Radiological assessments

Radiological evaluations were performed with total-body computed tomography (CT) scans every three months. Tumor response was assessed using RECIST version 1.1 (Response Evaluation Criteria in Solid Tumors), and findings were reviewed during multidisciplinary tumor board meetings.

### Genomic profiling and liquid biopsy

Molecular characterization was performed using FoundationOne® Liquid CDx (Foundation Medicine, Inc., Cambridge, MA), an FDA-approved assay for comprehensive genomic profiling of ctDNA. This test identifies SNVs, copy number variations, gene rearrangements, and genomic signatures such as TMB and MSI. TMB was reported as mutations per megabase (Muts/Mb). In this case, a *POLE P286R* mutation was identified in the proofreading exonuclease domain, consistent with an ultramutated phenotype. Tissue NGS analysis confirmed additional alterations in genes including *BRCA2, ATM, TP53,* and others.

### Treatment approach

Therapeutic decisions, including the initiation of off-label pembrolizumab, were made in a multidisciplinary context based on disease progression, molecular findings, and limited standard treatment options. All treatments were administered according to institutional protocols and guidelines. Ethical compliance The patient provided written informed consent for off-label treatment administration and for the publication of anonymized clinical data and images.

## Data Availability

The datasets generated and/or analyzed in this study are available from the corresponding author upon reasonable request.

## References

[1] Giunta EF et al (2020) Optimal treatment strategy for metastatic melanoma patients harboring BRAF V600 mutations. Ther Adv Med Oncol 12:1758835920915302. 10.1177/1758835920915302 32612709 10.1177/1758835920925219 PMC7307282

[2] Long GV et al (2015) Dabrafenib and trametinib versus dabrafenib and placebo for Val600 *BRAF*-mutant melanoma: a multicentre, double-blind, phase 3 randomised controlled trial. Lancet 386(9992):444–451. 10.1016/S0140-6736(15)60898-4 26037941 10.1016/S0140-6736(15)60898-4

[3] Liu L et al (2015) The BRAF and MEK inhibitors dabrafenib and trametinib: effects on immune function and in combination with immunomodulatory antibodies targeting PD-1, PD-L1, and CTLA-4. Clin Cancer Res 21(7):1639–1651. 10.1158/1078-0432.CCR-14-2339 25589619 10.1158/1078-0432.CCR-14-2339

[4] Kuske M et al (2018) Immunomodulatory effects of BRAF and MEK inhibitors: implications for Melanoma therapy. Pharmacol Res 136:151–159. 10.1016/j.phrs.2018.08.019 30145328 10.1016/j.phrs.2018.08.019

[5] Garmezy B et al (2022) Clinical and molecular characterization of *POLE* mutations as predictive biomarkers of response to immune checkpoint inhibitors in advanced cancers. JCO Precis Oncol 6:e2100267. 10.1200/PO.21.00267 35108036 10.1200/PO.21.00267 PMC8820927

[6] Siravegna G et al (2017) Integrating liquid biopsies into the management of cancer. Nat Rev Clin Oncol 14(9):531–548. 10.1038/nrclinonc.2017.14 28252003 10.1038/nrclinonc.2017.14

[7] Sweeney CJ et al (2015) Chemohormonal therapy in metastatic hormone-sensitive prostate cancer. N Engl J Med 373(8):737–746. 10.1056/NEJMoa1503747 26244877 10.1056/NEJMoa1503747 PMC4562797

[8] De Bono JS et al (2011) Abiraterone and increased survival in metastatic prostate cancer. N Engl J Med 364(21):1995–2005. 10.1056/NEJMoa1014618 21612468 10.1056/NEJMoa1014618 PMC3471149

[9] Le DT et al (2015) PD-1 blockade in tumors with mismatch repair deficiency. N Engl J Med 372(26):2509–2520. 10.1056/NEJMoa1500596 26028255 10.1056/NEJMoa1500596 PMC4481136

[10] Zhao P et al (2019) Mismatch repair deficiency/microsatellite instability-high as a predictor for anti-PD-1/PD-L1 immunotherapy efficacy. J Hematol Oncol 12(1):54. 10.1186/s13045-019-0738-1 31151482 10.1186/s13045-019-0738-1 PMC6544911

[11] Brahmer JR et al (2012) Safety and activity of anti–PD-L1 antibody in patients with advanced cancer. N Engl J Med 366(26):2455–2465. 10.1056/NEJMoa1200694 22658128 10.1056/NEJMoa1200694 PMC3563263

[12] Reichert ZR et al (2019) Microsatellite instability as an emerging biomarker for checkpoint inhibitor response in advanced prostate cancer. JAMA Oncol 5(4):478–479. 10.1001/jamaoncol.2018.6760 30589921 10.1001/jamaoncol.2018.5789

[13] Graham LS et al (2020) Mismatch repair deficiency in metastatic prostate cancer: response to PD-1 blockade and standard therapies. PLoS ONE 15(6):e0233260. 10.1371/journal.pone.0233260 32453797 10.1371/journal.pone.0233260 PMC7250457

[14] Hoch D, Rabaglio M, Grob T, von Gunten M, Beyer J, Akhoundova D (2023) Exceptional response to pembrolizumab in a mismatch repair-deficient aggressive prostate cancer with somatic EPCAM, MSH2, and *MSH6* co-deletion: a case report. Case Rep Oncol 16(1):1280–1286. 10.1159/000534177 37928863 10.1159/000534177 PMC10622161

[15] Dinerman BF, Skomra A, Dovirak I, Rutkowski J (2024) Utility of pembrolizumab for metastatic castrate resistant prostate cancer with MMR deficiency. Urology Case Rep 57:102833. 10.1016/j.eucr.2024.102833 10.1016/j.eucr.2024.102833 PMC11408038 39301117

[16] Lambert N, Moore C, Klavon J, Arafat W, Wang J, Zhang T, Courtney K (2025) Clinical outcomes of patients with metastatic prostate cancer with microsatellite instability treated with pembrolizumab. Clin Genitourin Cancer 23(5):102384. 10.1016/j.clgc.2025.102384 40700962 10.1016/j.clgc.2025.102384

[17] Fujiwara M, Komai Y, Yuasa T, Numao N, Yamamoto S, Fukui I, Yonese J (2020) Pembrolizumab for a patient with metastatic castration-resistant prostate cancer with microsatellite instability-high. IJU Case Rep 3(2):62–64. 10.1002/iju5.12144 32743472 10.1002/iju5.12144 PMC7292086

[18] Shiba K, Fujiwara M, Onuki A, Kato D, Shirakawa T, Shimizu Y, Amemiya T, Nenohi T, Matsumoto Y, Urushibara M, Kano H, Ishizaka K, Takahashi M, Yokoyama M (2025) Pembrolizumab for treatment-related neuroendocrine prostate carcinoma with a high tumor mutational burden: a case report. Front Oncol 15:1642412. 10.3389/fonc.2025.1642412 40823094 10.3389/fonc.2025.1642412 PMC12355211

[19] Muraoka S, Tosuji H, Iwahashi Y, Kawabata H, Deguchi R, Wakamiya T, Yamashita S, Kohjimoto Y, Hara I (2025) TMB-high, MSI-high castration-resistant prostate cancer treated with pembrolizumab. IJU Case Rep 8(5):449–453. 10.1002/iju5.70062 40909311 10.1002/iju5.70062 PMC12408169

